# Isolated cryptococcal osteomyelitis of the sacrum in an immunocompetent patient: a case report and literature review

**DOI:** 10.1186/s12879-023-08066-6

**Published:** 2023-02-24

**Authors:** Yanchun Zhong, Yuxi Huang, Di Zhang, Zhaoyuan Chen, Zhenxing Liu, Yongjun Ye

**Affiliations:** 1 Department of Orthopaedics, First Affiliated Hospital of Gannan Medical University, Ganzhou, 341000 People’s Republic of China; 2 Department of Basic Medicine, Gannan Healthcare Vocational College, Ganzhou, 341000 People’s Republic of China; 3 Department of Medical Imaging, First Affiliated Hospital of Gannan Medical University, Ganzhou, 341000 People’s Republic of China

**Keywords:** *Cryptococcus neoformans*, Sacrum, Cryptococcal osteomyelitis, Fungus, Infection

## Abstract

**Background:**

*Cryptococcus neoformans*, an opportunistic fungal pathogen, seldom causes infection in immunocompetent people. Cryptococcal osteomyelitis is an uncommon condition in which *Cryptococcus* invades the bone. It usually occurs as part of a disseminated infection and rarely in isolation. The spine has been reported as the most common site of cryptococcal osteomyelitis; however, isolated case of sacrum involvement in immunocompetent patients has never been reported.

**Case presentation:**

We report the case of a 37-year-old man without underlying disease who presented with progressive low back and sacrococcygeal pain. The patient was initially diagnosed with sacral tumour by a local doctor, and subsequently, after admission, was diagnosed with sacral tuberculosis. He was empirically treated with antitubercular drugs. The patient failed to respond to antitubercular drugs and complained of worsening low back pain. Additionally, he developed persistent radiating pain and numbness in his legs. For further diagnosis, we performed a computed tomography-guided puncture biopsy of the sacrum, which revealed granulomatous inflammation with massive macrophage infiltration and special staining revealed a fungal infection. We performed sacral debridement and drainage and obtained purulent specimens for pathological examination and microbial culture. Microbial identification and drug susceptibility tests revealed a *Cryptococcus neoformans* infection sensitive to fluconazole. Postoperatively, the persistent radiating pain and numbness in the legs resolved. After 12 consecutive weeks of antifungal therapy, all his symptoms resolved. The patient remained without any signs of recurrence at the 8-month follow-up.

**Conclusion:**

We reported a rare case of isolated sacrum cryptococcal osteomyelitis in an immunocompetent patient. Furthermore, we identified and reviewed 18 published cases of spine cryptococcal osteomyelitis. Immunocompetent individuals are also at risk for cryptococcal osteomyelitis. Clinical manifestation and imaging are insufficient to diagnose cryptococcal osteomyelitis of the spine, and invasive examinations, such as puncture biopsy and fungal examinations, are needed. Antifungal therapy yields satisfactory results for the treatment of cryptococcal osteomyelitis of the spine, however, if the infective lesion is large, especially when it compresses the spinal cord and nerves, a regimen combining aggressive surgery with antifungal therapy is indispensable.

## Background

*Cryptococcal neoformans*, an opportunistic invasive fungus, is abundant in topsoil, rotten food, and bird droppings, especially pigeon droppings [1–3]. As the most common etiologic agent of cryptococcosis, *Cryptococcus neoformans* seldom causes infection in immunocompetent people and mainly afflicts immunocompromised hosts, such as patients with acquired immune deficiency syndrome (AIDS), lymphoma, organ transplantation, tuberculosis, or patients undergoing steroid therapy [4, 5]. As reported in previous studies, the incidence of cryptococcosis is about 5–10% in immunocompromised patients and 30% in AIDS patients, but only one in 100,000 in immunocompetent people [1, 4].

Generally, *Cryptococcus* invades through the respiratory tract [6]. After inhalation, *Cryptococcus* can localize in the lungs and cause cryptococcal pneumonia or disseminate hematogenously, affecting other extrapulmonary infection sites [7]. Although the lungs and central nervous system are the most commonly affected sites, other organs or tissues may be infected [2, 3, 8]. Occasionally, the skeleton can also be invaded leading to cryptococcal osteomyelitis, which usually results from the hematogenous spread of lung infection [3, 9]. Cryptococcal osteomyelitis is uncommon, especially in immunocompetent people, comprising only 5% of all cryptococcal infections [5, 10, 11]. Cryptococcal osteomyelitis usually occurs as part of a disseminated infection and rarely in isolation. Though spine vertebrae have been reported to be the most common site of cryptococcal osteomyelitis, reports on sacral vertebrae involvement are scarce [12–14].

Herein, we report a case of isolated cryptococcal osteomyelitis of the sacrum in an immunocompetent patient and review the literature on cryptococcal osteomyelitis involving the spine in the last 30 years. We hope these studies can help other clinicians manage similar cases.

## Case presentation

A 37-year-old man presented to the Department of Oncology in our hospital with a 3-month history of progressive low-back and sacrococcygeal-pain, with occasional pain radiating bilaterally to the legs. He did not have fever, headaches, cough, or night sweats. Additionally, he had no history of AIDS, diabetes, tuberculosis, leukaemia, lymphoma, or immunosuppressive treatment. He initially visited a local county hospital, where lumbar vertebra magnetic resonance imaging (MRI) was performed. Findings showed osteolytic lesions of the S2–S4 vertebral body accompanied by paravertebral soft tissue masses, with mixed hyperintense signal on T2-weighted imaging (T2WI), and low signal intensity on T1-weighted imaging (T1WI). His preliminarily diagnosis was a sacral tumour, and he was referred to another hospital for further treatment.

**Fig. 1:**
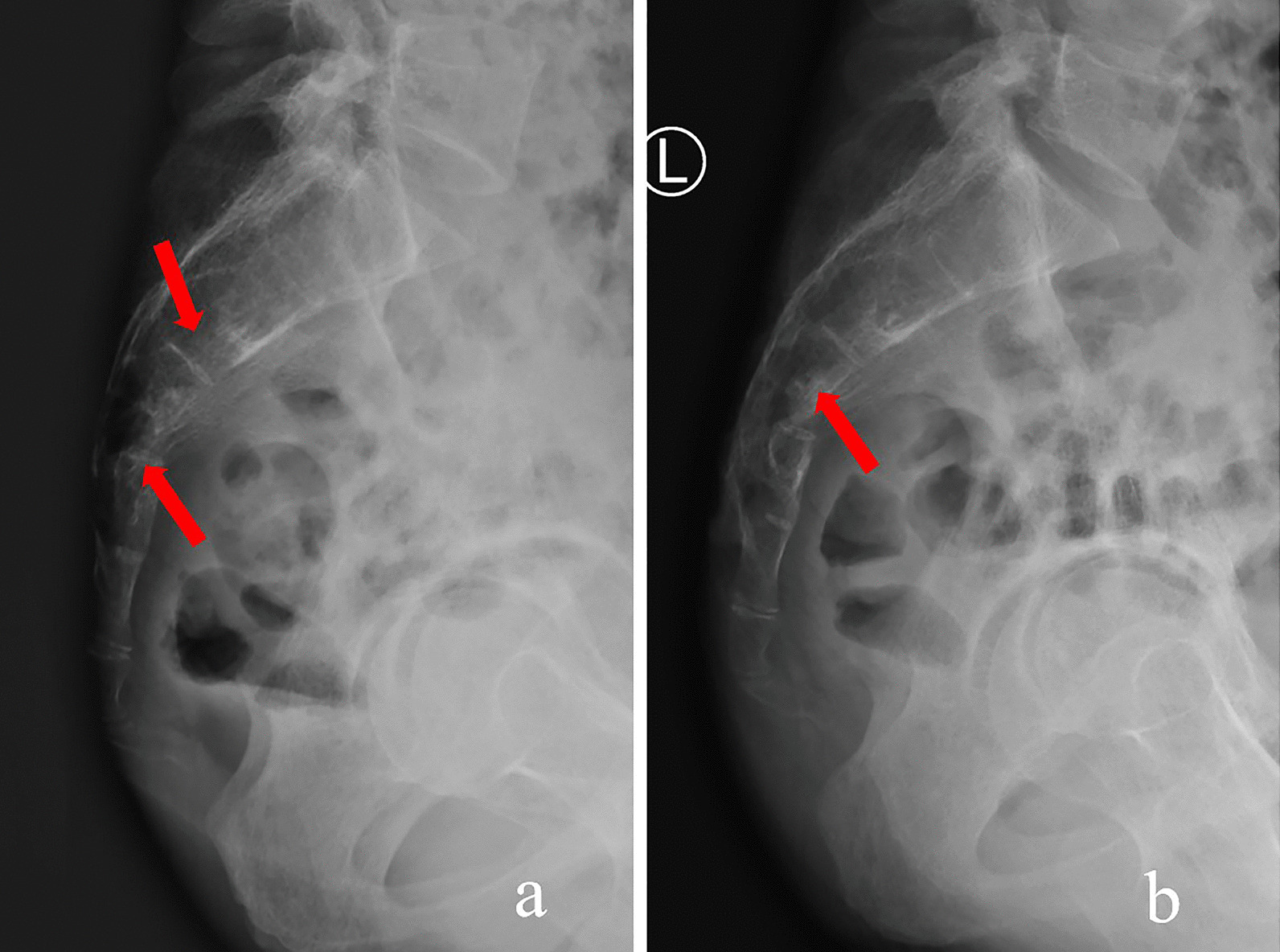
Lateral radiography (sacrum). **a **Preoperative radiograph: patchy osteolytic lesions in S2–S4 with an unclear boundary (red arrows). **b **Radiograph, 6-month postoperatively: bone defect lesions of the sacrum with clear boundary and rim sclerosis (red arrows)

**Fig. 2:**
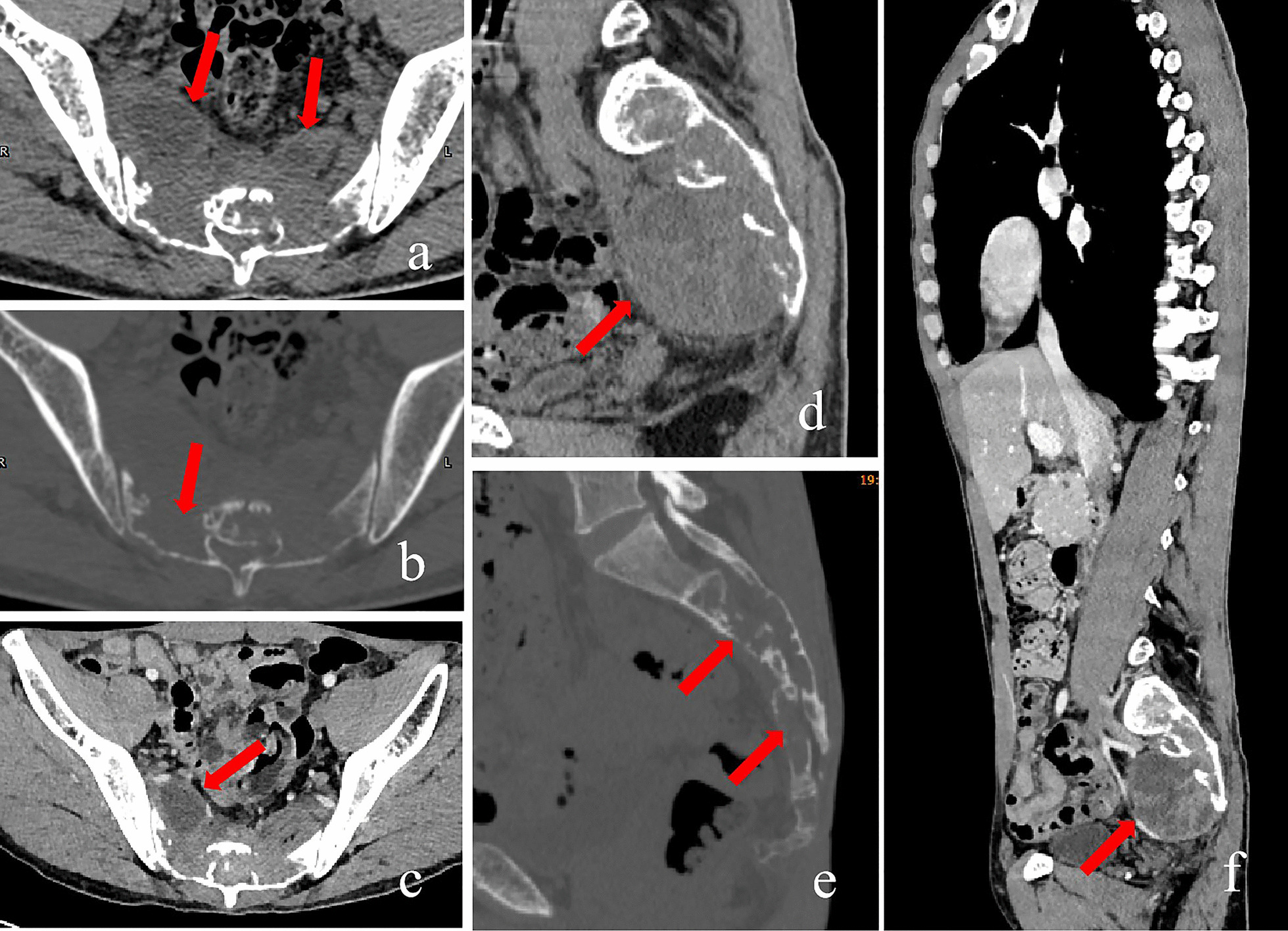
Preoperative computed tomography (CT) (sacrum). Non-enhanced CT scan (**a** transversal soft tissue window; **b **transversal bone window; **d **sagittal soft tissue window; **e **sagittal bone window): osteolytic lesions surrounded by cystic low-density soft-tissue masses of unequal-size, largest in anterior-right (red arrows). Contrast-enhanced CT (**c **transversal; **f **sagittal): soft-tissue masses with heterogeneous ring enhancement and unremarkable central enhancement (red arrows)

**Fig. 3:**
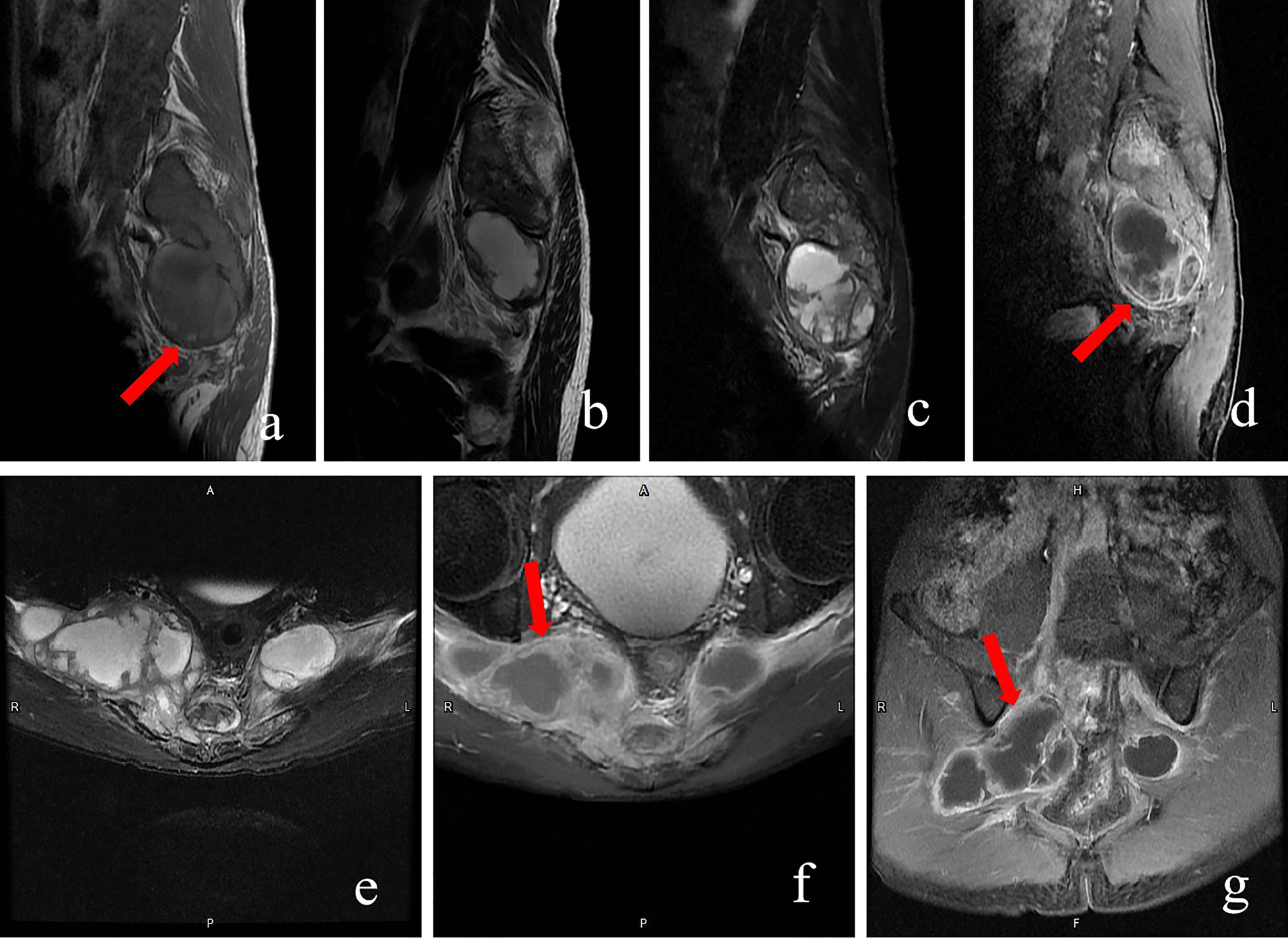
Preoperative magnetic resonance imaging (MRI) (sacrum). MRI (**a **sagittal T1-weighted; **b **sagittal T2-weighted; **c** sagittal fat-suppressed T2-weighted; **e**:transversal fat-suppressed T2-weighted): patchy abnormal signals in the sacrum and attached soft-cystic masses in the presacral, hyperintense signal on T2-weighted imaging (T2WI), and low-signal intensity on T1-weighted imaging (T1WI) (red arrows). Contrast-enhanced MRI (**d** sagittal enhanced T1-weighted; **f **transversal enhanced T2-weighted; **g **coronal enhanced T2-weighted): pre-sacral soft-tissue masses with thickened and enhanced soft cystic wall (red arrows)

**Fig. 4:**
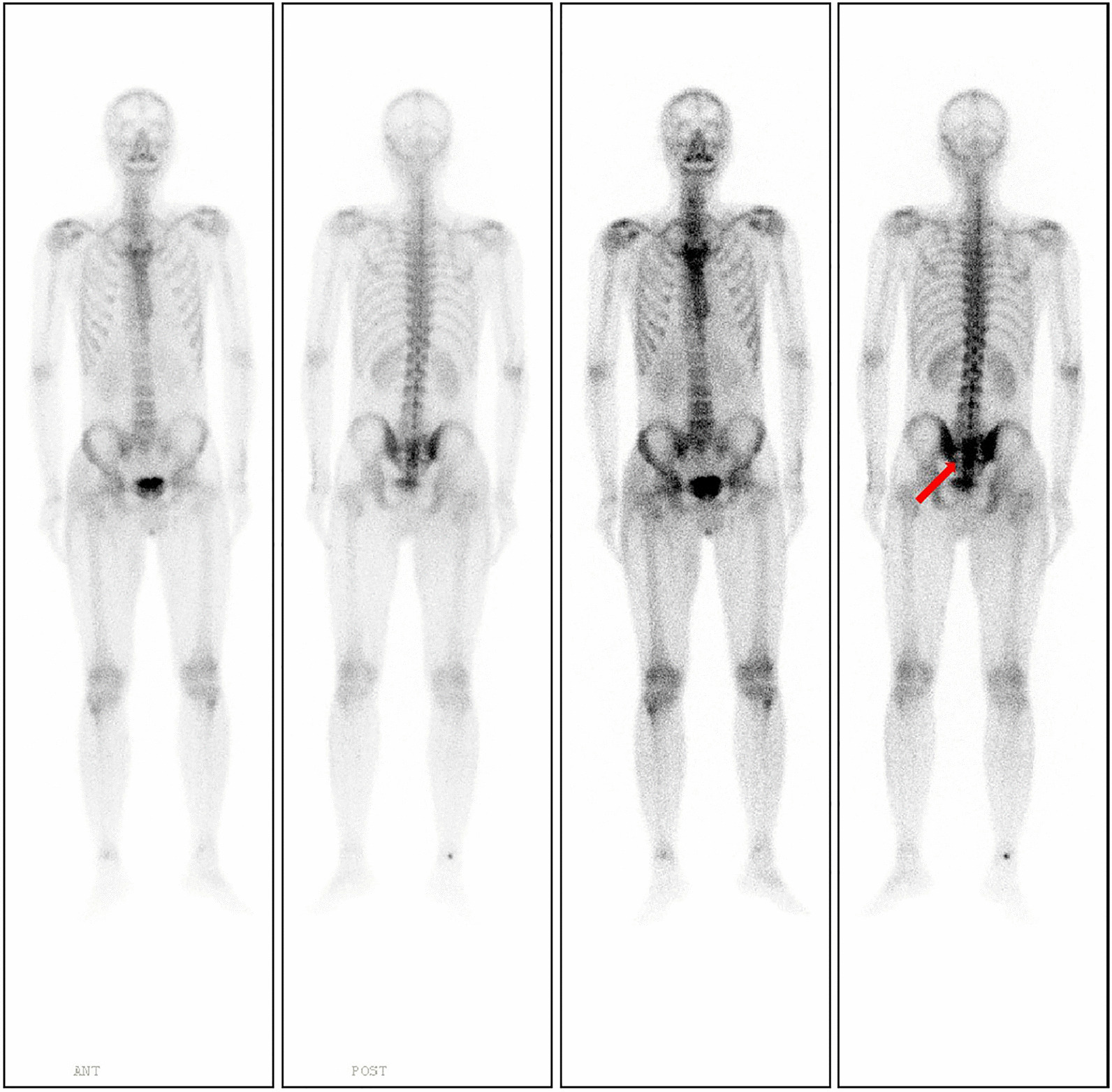
Systemic radionuclide bone-scanning shows active metabolism of sacrum (red arrows)

His clinical examination on admission revealed local tenderness and percussion pain in the low back and sacrococcygeal region. When tapping the lumbosacral regions, pain occasionally radiated to both legs, particularly the right side. The lumbar spine’s movement was normal, with no abnormal strength or sensation in both lower extremities. Routine blood tests revealed white blood cell count of 11.2 × 10^9^/L (normal 3.5–9.5 × 10^9^/L) with 88% neutrophils (normal 40–75%), 6% lymphocytes (normal: 20–50%), and 0.2% eosinophils (normal: 0.4–8%). Erythrocyte sedimentation rate (ESR) was 22 mm/h (normal 0–20 mm/h) and C-reactive protein (CRP) level was 116.98 mg/L (normal 0–6.0 mg/l). Tumour markers were not elevated, and all other test results were normal. Radiographs revealed a patchy osteolytic lesion in S2–S4 with an unclear boundary, without periosteal reaction and rim sclerosis (Fig. 1a). Computed tomography (CT) of the sacrococcygeal vertebra showed some irregular osteolytic lesions in the sacrum, which were surrounded by several cystic low-density soft tissue masses of unequal size, the larger one in the anterior-right was approximately 52 × 84 mm (Fig. 2). MRI illustrated abnormal patchy signals in the sacrum, hyperintense signal on T2WI and low signal intensity on T1WI (Fig. 3a, b). Moreover, attached cystic soft masses were seen in the presacral space (Fig. 3). A contrast-enhanced fat-suppressed T2-weighted MRI demonstrated a thickened and enhanced soft cystic wall (Fig. 3d, f, g). Except for sacral lesions, no other abnormalities were found on the contrast-enhanced CT of the chest and whole abdomen. A radioisotope bone scan illustrated increased tracer uptake in the sacrum (Fig. 4).

**Fig. 5:**
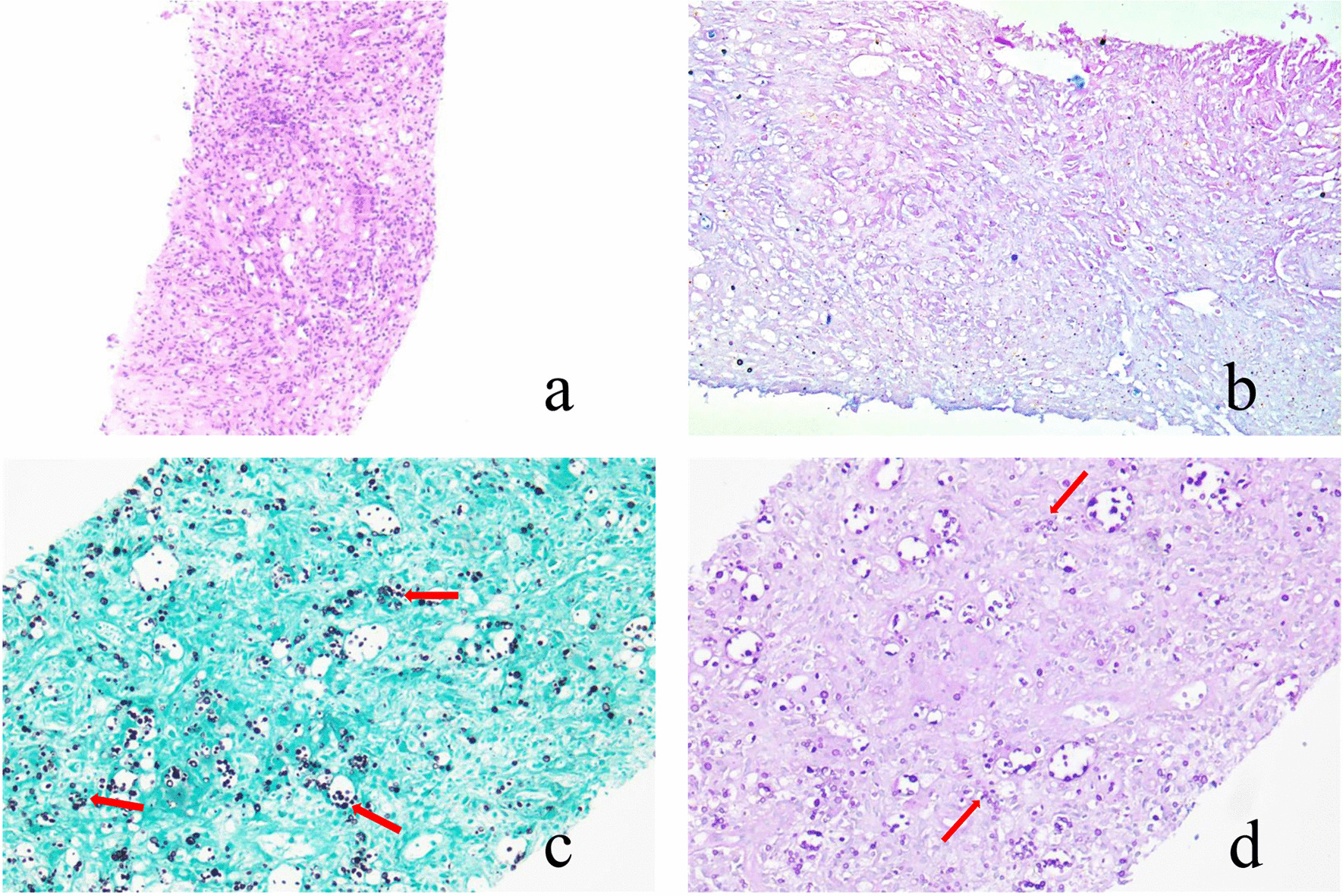
Puncture biopsy-sample with staining methods. **a** Hematoxylin-Eosin shows granulomatous inflammation with massive macrophage infiltration; **b** Acid fast is negative; **c** Grocott’s methenamine silver shows numerous black stained, small yeast-like organisms (red arrows); **d **Periodic acid-Schiff shows large number of spheroids with red-outer-membranes of various sizes (red arrows)

Imaging and blood test results were characteristic of inflammatory changes and suggestive of sacral tuberculosis. Therefore, the patient was transferred to the Department of Orthopaedics for further treatment. He received a four-drug combination antitubercular therapy. After about one week of antitubercular therapy, the low back and sacrococcygeal pain worsened, and the patient developed persistent radiating pain and numbness in the legs, and night-time fever (38.3–39.5 ℃). For further diagnosis, we performed a CT-guided puncture biopsy of the sacral lesion and a blood culture. Hematoxylin and Eosine staining of pathologic samples revealed granulomatous inflammation with massive macrophage infiltration (Fig. 5a). Acid-fast staining was negative (Fig. 5b) and Grocott’s methenamine silver staining showed numerous black stained, small yeast-like organisms (Fig. 5c). Periodic acid-Schiff staining showed a large number of spheroids with red outer membranes of varying sizes (Fig. 5d). Based on these pathological findings, fungal infection was highly suspected. However, as the blood culture was negative, we did not perform microbial culture on the puncture specimen at that time. To identify the fungal species, we conducted a galactomannan detection test (GM-test) and tried to detect cryptococcal antibodies in serum; the GM-test result was normal. Owing to facility issues, we could not complete the cryptococcal antibody test.

**Fig. 6:**
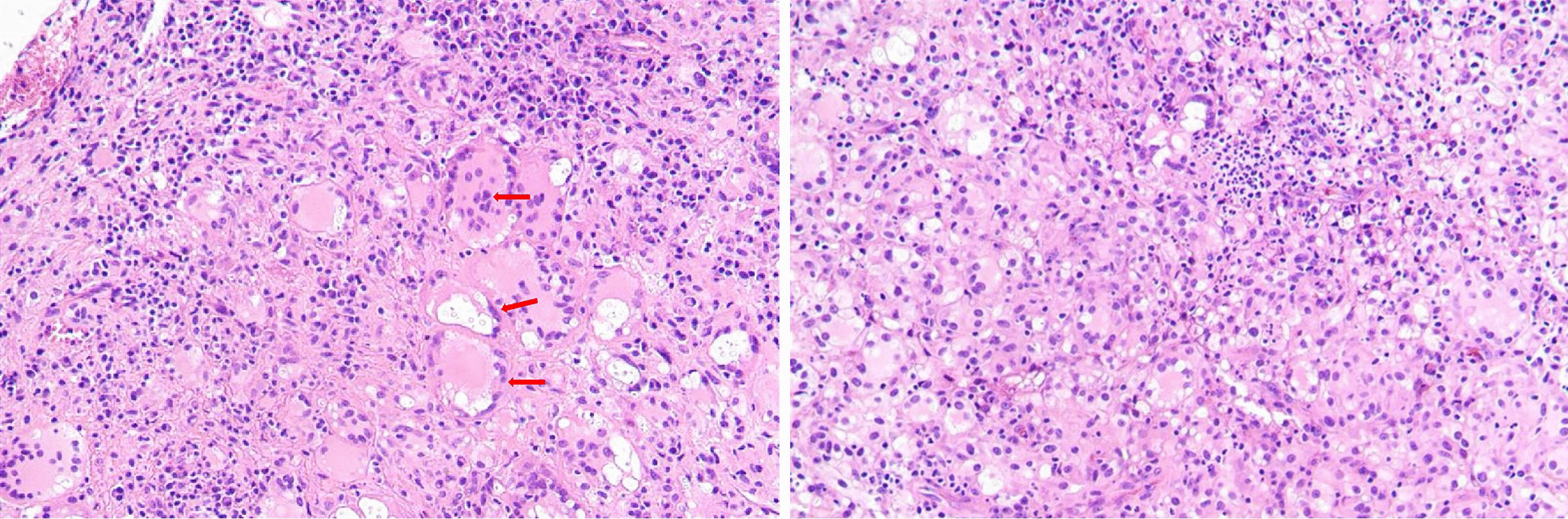
Postoperative pathologic examination showing granuloma formation containing large quantities of multinucleated giant-cells, with round, slightly stained, thin-walled *Cryptococcus* in cytoplasm, surrounding transparent voids, and partial budding (red arrows)

**Fig. 7:**
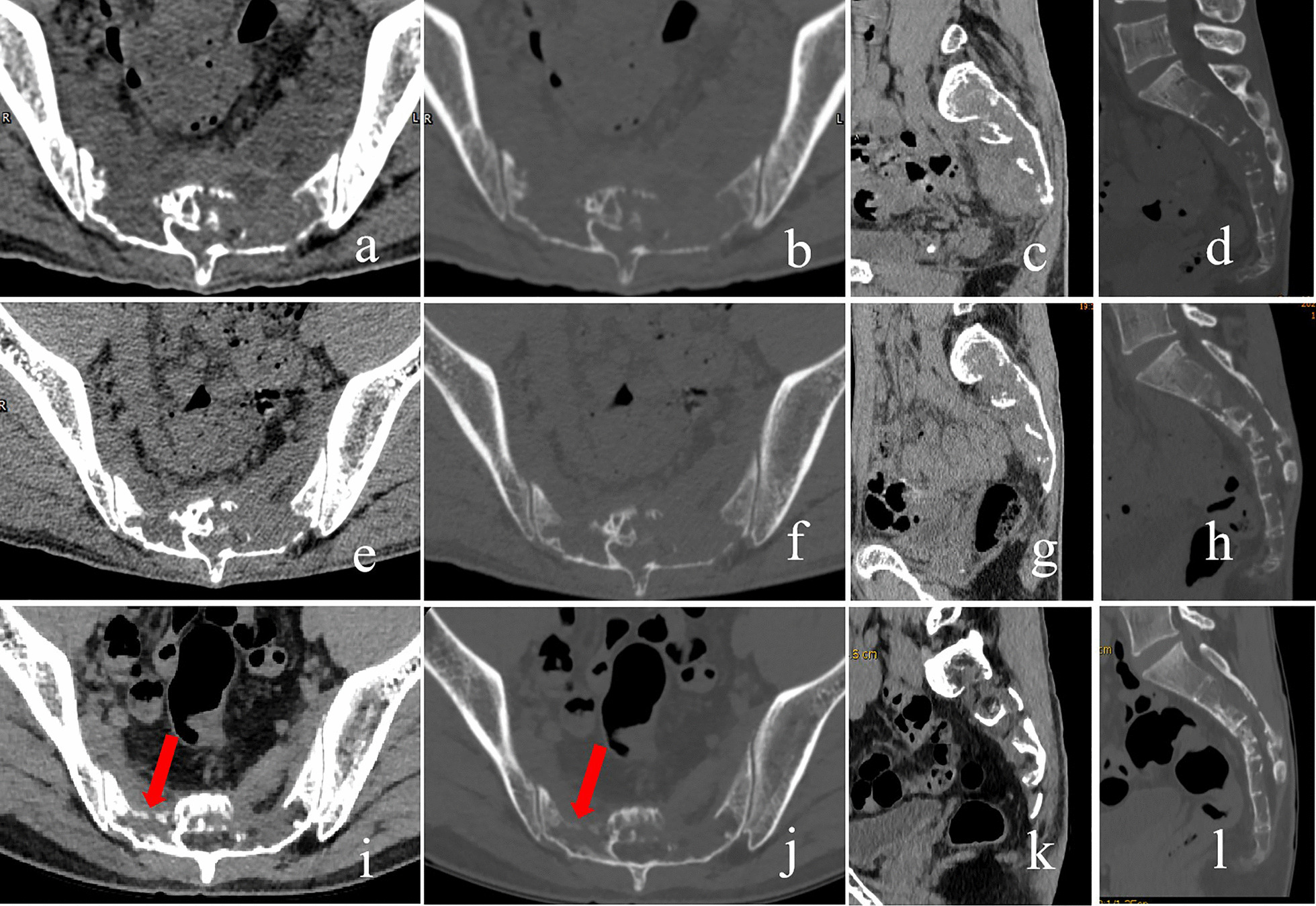
Postoperative computed tomography (CT) (sacrum). One-month postoperative CT (**a** transversal soft tissue window; **b** transversal bone window; **c** sagittal soft tissue window; **d** sagittal bone window) -abscess became smaller, and bone defect lesions show no changes. Three-month post-operative CT (**e** transversal soft tissue window; **f **transversal bone window; **g** sagittal soft tissue window; **h** sagittal bone window): no abscess, bone defect lesions became smaller. Six-month post-operative CT (**i **transversal soft tissue window; **j **transversal bone window; **k **sagittal soft tissue window; **l** sagittal bone window): new bone on edge of bone defect lesions (red arrows) and no relapse

**Fig. 8:**
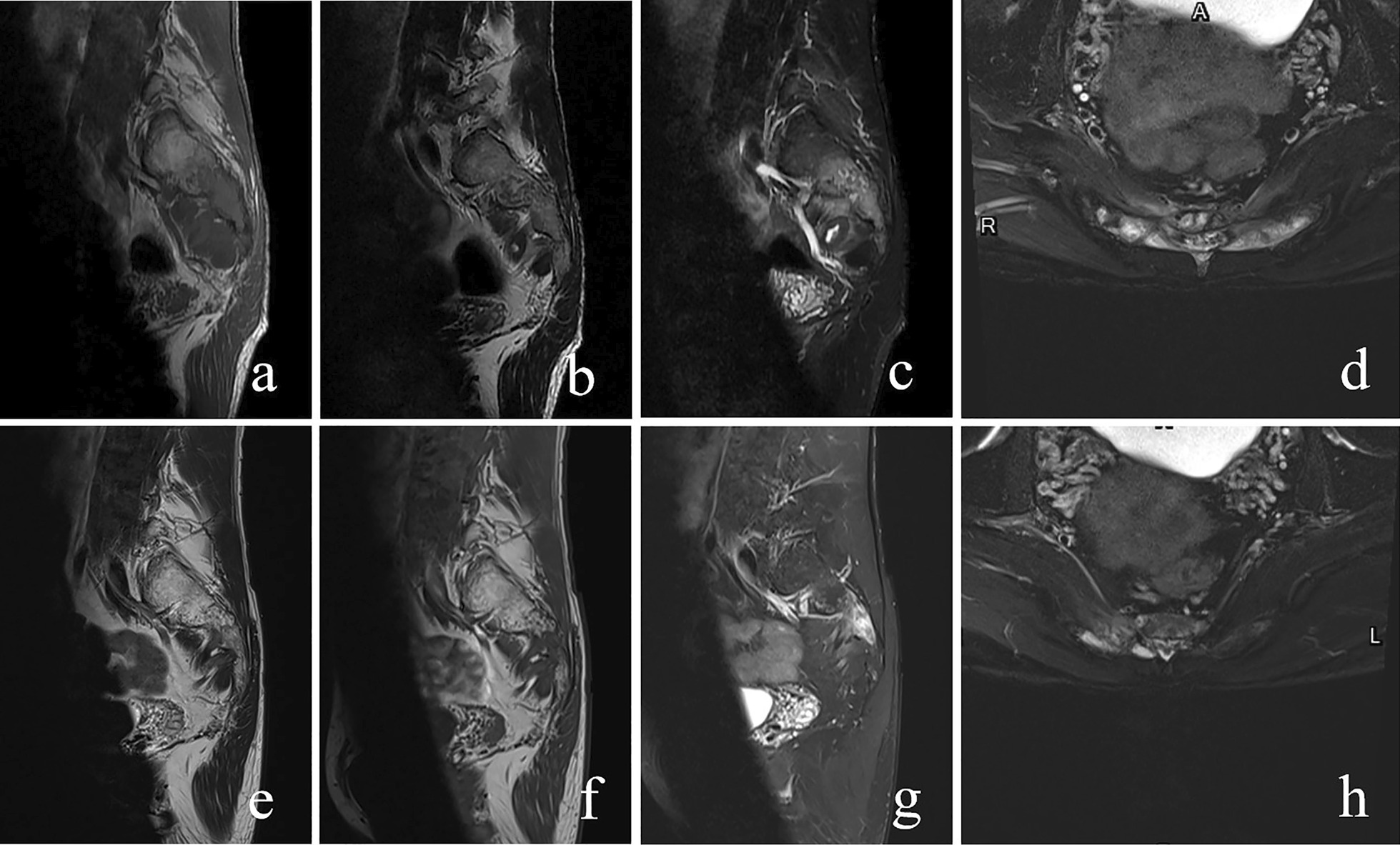
Postoperative magnetic resonance imaging (MRI)(sacrum). One-month post-operative MRI (**a** sagittal T1-weighted; **b **sagittal T2-weighted; **c** sagittal fat-suppressed T2-weighted; **d** transversal fat-suppressed T2-weighted ): abscess became smaller. Six-month post-operative MRI (**e** sagittal T1-weighted; **f** sagittal T2-weighted; **g** sagittal fat-suppressed T2-weighted; **h** transversal fat-suppressed T2-weighted ): abscess resolved

The patient underwent surgical sacral debridement and negative drainage through the anterior approach under intravenous titration of fluconazole. Intraoperatively, we observed that the anterior cortical bone of the sacrum had multi-point destruction, and the lesion was unequal in size and depth. Moreover, several honeycombed abscesses filled with weak pus were observed in the presacral space. The sacral nerve roots were compressed by cystic abscesses. Samples from the abscesses were sent to the laboratory for pathological examination, microbial culture, identification, and drug susceptibility tests. Postoperatively, the persistent radiation pain and numbness in the legs resolved. The postoperative pathological examination revealed granuloma formation containing large quantities of multinucleated giant cells, with some round, slightly stained, thin-walled *Cryptococcus* in the cytoplasm, surrounding transparent voids, and partial budding (Fig. 6). Moreover, the sample was inoculated in Sabouraud Dextrose Agar supplemented with chloramphenicol (0.5 g/l). After 5 days of culture at 37 ℃, yellow colony growth was observed, in which microscopic examination showed round or oval thalli with budding cells but without pseudo mycelium. The pathogen was identified as *Cryptococcus neoformans* using the BRUKER MALDI-TOF Biotyper mass spectrometry system. Antifungal susceptibility testing performed using ATB FUNGUS 3 system revealed sensitivity to fluconazole. Ultimately, the patient was definitively diagnosed with *Cryptococcal neoformans* sacral osteomyelitis and was treated with fluconazole for 12 weeks (4 weeks of intravenous fluconazole 400 mg/day, then 8 weeks of oral fluconazole, 400 mg/day). At the follow-up, the low back and sacrococcygeal pain had progressively alleviated. Six months after surgery, his back pain completely resolved, and his ESR, CRP level, and blood cell count were normal. The patient recovered completely without radiological evidence of relapse at the 8-month follow-up (Figs. 1b, 7 and 8).

## Discussion and conclusions

Cryptococcosis in immunocompetent people is infrequent, and isolated cryptococcal osteomyelitis is extremely rare [13, 15, 16]. The spine vertebrae are considered the most common site of cryptococcal osteomyelitis [12–14]; however, its incidence remains low, and the preferred section of the spine for *Cryptococcus* infection remains unclear. On reviewing all English language reports of cryptococcal osteomyelitis involving the spine since 1992, we identified 18 related cases (Table 1) [1–7, 10, 12, 17–25]. Among them, 10 (55.6%) cases involved the lumbar vertebrae, 7 (38.9%) the thoracic vertebrae, 3 (15.8%) the sacral vertebrae, and none were reported in the cervical or coccygeal vertebrae. This suggests that the lumbar vertebrae are the most common sites of cryptococcal osteomyelitis in spine.

**Table 1:** Main characteristics of 18 published cases of spine cryptococcal osteomyelitis

Author	Age (years)/sex	Site	Comorbidities	Initial diagnosis	Diagnosis techniques	Isolated or disseminated	Surgery	Antifungal drug	Method of medication	Course of medication	Follow up	Clinical outcome
Adsul et al.[1]	44/F	T4	Diabetes	Tuberculosis	Postoperative culture and histopathological examinations revealed cryptococcal infection	Isolated	T4 decompression with pedicle screw fixation	Amphotericin B Fluconazole Flucytosine	3 months of intravenous amphotericin B and flucytosine postoperative, then 5 months of oral fluconazole and flucytosine	8 months	8 months	Improvement
Al-Tawfiq and Ghandour [4]	34/F	L4	Tuberculous lymphadenitis	Pyogenic osteomyelitis	Abscess culture grew *C. neoformans*. The serum cryptoccocal antigen (-)	Isolated	The vertebral abscess was drained and aspirated	Fluconazole	12 weeks of oral fluconazole	12 weeks	12 months	Full recovery
Joo et al.[2]	66/F	L2	Rectal cancer with adjuvant chemotherapy	Metastatic tumor	Postoperative pathologic examination. CSF culture and cryptococcal antigen (-)	Isolated	Corpectomy of L2 vertebral body	Amphotericin B Fluconazole	1 week of intravenous amphotericin B and fluconazole, then, 1 year of oral fluconazole	12 months	12 months	Full recovery
Lai et al. [20]	25/M	L1, S1	No	Osteosarcoma	Preoperative biopsy revealed an infected lesion, postoperative microbial identification revealed cryptococcal infection	Disseminated	Lumbosacral debridement	Amphotericin B	4 weeks of intravenous amphotericin B and then 8 weeks of oral amphotericin B	12 weeks	9 months	Full recovery
Li et al. [17]	17/F	L1	Immune hemolytic anemia	Tuberculosis	*C. neoformans* was isolated in CSF cultures. CSF cryptococcus antigen (+), Preoperative specific stains for *C. neoformans* (+)	Isolated	Spinal debridement and fusion	Fluconazole	3 months of oral fluconazole	Unclear	3 months	Full recovery
Nankeu et al. [10]	29/M	S1,S2	Chronic hepatitis B	Cryptococcal infection	Blood cultures and biopsy specimen were positive for *C. neoformans.* Cerebrospinal fluid *C.neoformans* antigen (+)	Disseminated	No	Amphotericin B, Fluconazole, Flucytosine	4 weeks of intravenous amphotericin B and flucytosine, followed by 18 months of oral fluconazole	19 months	2 years	Full recovery
Noh et al. [21]	21/F	The sacrum	Autoimmune hepatitis with chronic steroid therapy	Cryptococcal infection	Cryptococcal antigen of serum and cerebrospinal fluid (+), Fungal culture (+)	Disseminated	Incision and debridement	Amphotericin B	Unclear	3 months	3 years	Full recovery
Wang et al. [5]	67/F	T2, T3	No	Cryptococcal infection	Preoperative serum cryptococcal antigen (+), Postoperative pathological examination(+)	Isolated	Lesion clearance followed by intramedullary nailing and allogeneic bone transplantation	Voriconazole Fluconazole	8 weeks of intravenous voriconazole and then 4 weeks of oral fluconazole	12 weeks	12 weeks	Full recovery
Wang et al.[6]	41/F	L4	No	Cryptococcal infection	Preoperative percutaneous biopsy(+), Postoperative Microbial culture(-) Next-generation sequencing	Disseminated	Posterior lumbar open-window focal debridement	Flucytosine, Amphotericin B, Fluconazole	Unclear	20 days	12 years	Full recovery
Zhou et al. [3]	40/F	L4	Rheumatoid arthritis and scleroderma	Cryptococcal infection	Needle aspiration biopsy(+), Microbial culture(+)	Isolated	No	Fluconazole	6 months of oral fluconazole	6 months	12 months	Full recovery
Gupta et al. [7]	42/F	T2, T3	Tuberculous lymphadenopathy	Tuberculosis	Postoperative histopathological examination	Isolated	T2, T3 costotransversectomy	Amphotericin B Flucytosine	Unclear	2 weeks	2 weeks	Death
Wildstein et al.[18]	20/M	T12-L2	Sarcoidosis with prednisone therapy	Cryptococcal infection	Cryptococcal antigen (-) Biopsy of the paraspinal mass histologically revealed the presence of fungal organisms	Disseminated	No	Fluconazole	Oral fluconazole	unclear	6 months	Full recovery
Cook [19]	24/F	T1-T3	Sarcoidosis with corticosteroids therapy	Cryptococcal infection	Needle aspiration biopsy(+), Microbial culture(+) Cryptococcal antigen (-)	Isolated	Percutaneous puncture drainage	Fluconazole, Flucytosine, Amphotericin B	12 months of oral antifungal drug	12 months	16 months	Full recovery
Gurevitz et al.[12]	67/F	L3	No	Cryptococcal infection	Open biopsy(+), Microbial culture(+) Cryptococcal antigen (+)	Isolated	No	Fluconazole, 5-fluorocytosine	6 weeks of intravenous amphotericin B and oral 5-fluorocytosine	6 weeks	2 years	Full recovery
Jain et al.[22]	72/F	T6	Diabetes	Tuberculosis	FNAP Fungal culture	Isolated	No	Flucytosine, Amphotericin B	3 months of intravenous amphotericin B and oral flucytosine	3 months	5 years	Full recovery
Glynn et al. [23]	52/F	L1-L3	No	Cryptococcal infection	Needle percutaneous biopsy showed chronic inflammation but culture revealed *C. neoformans*	Isolated	No	Amphotericin B 5- Fluorocytosine Ketoconazole	6 weeks of intravenous amphotericin B and oral 5-fluorocytosine and then 18 weeks of oral ketoconazole	24 weeks	7 years	Full recovery
Ruan et al. [24]	68/M	T5-T11	No	pyogenic infection Tuberculosis	Cultures of blood and percutaneous aspiration culture of pus revealed *C. neoformans*	Disseminated	Excision of paravertebral abscess	Amphotericin B Itraconazole Fluconazole Fluorocytosine	Starting with intravenous amphotericin B and itraconazole, followed by oral fluconazole and fluorocytosine	4 months	18 months	Improvement
Xu et al. [25]	42/F	L1-L5	Sjögren’s syndrome and treated with methylprednisolone	Cryptococcal infection	Tissue biopsies granulomatous lesions with visible cryptosporidium	Disseminated	No	Fuconazole	6 months of oral fluconazole	6 months	1 years	Full recovery

We found only three published cases of cryptococcal osteomyelitis involving the sacrum [10, 20, 21]. Lai et al. [20] reported a case of nonadjacent cryptococcal infection involving L1 and S1 vertebrae. The other two cases by Nankeu et al. and Noh et al. reported disseminated cryptococcosis involving the sacrum and the patients had underlying comorbidities [10, 21]. To our knowledge, this is the first report of isolated cryptococcal osteomyelitis of the sacrum in an immunocompetent patient.

Common clinical manifestations of spine cryptococcal osteomyelitis include local pain, tenderness, and oedema, sometimes accompanied by fever, weakness, and other manifestations of spinal cord compression [6, 26]. Imaging findings usually comprise irregular osteolytic destruction of the vertebral bodies with or without paraspinal abscess [7, 12, 13]. Numerous diseases, such as tumours and bone tuberculosis, have similar manifestations and imaging findings. Due to the low incidence, atypical manifestations, and nonspecific imaging findings, spine cryptococcal osteomyelitis is a diagnostic challenge and is easily misdiagnosed, delaying treatment in many cases [5, 21]. In our literature review, eight (44.4%) cases were initially misdiagnosed as bone tuberculosis, malignant neoplasm, or pyogenic osteomyelitis, and then were finally diagnosed correctly by pathological and microbial culture examinations after inappropriate surgery or drug treatment.

Our patient was a young man without underlying disease and had no history of abnormal immune function. He was an immunocompetent individual, and not in a susceptible population. His main symptom was low back pain and clinical examination revealed local tenderness and percussion pain. His blood tests were nonspecific, only showing elevated inflammatory markers. In addition to bone destruction, his imaging findings revealed sizeable soft tissue masses around the sacrum, similar to some primary sacral tumours, such as chordoma and giant cell tumours of the bone. Therefore, he was misdiagnosed with tumour and bone tuberculosis. Finally, an accurate diagnosis was made by a timely puncture biopsy and postoperative microbial culture.

The confirmative diagnosis of cryptococcal osteomyelitis relies on positive culture and histological examination of the infective lesion specimens [6, 7, 21]. Hence, obtaining the infective lesion specimen is critical for diagnosis [15, 24, 27]. It is easy to accomplish this in a superficial lesion or ruptured abscess, but not in spine cryptococcal osteomyelitis cases where lesions are usually deep, and the abscess rarely ruptures outwards [15, 24, 26]. Puncture biopsy, an invasive examination for obtaining a specimen, is generally performed under the guidance of CT, which was associated with less trauma and high accuracy in diagnosing spine cryptococcal osteomyelitis in some cases [3, 10, 16, 20, 25]. In half of the cases reviewed by us, the specimens were obtained by puncture biopsy, and diagnosis was confirmed by fungal culture and pathological examination of the specimen. Our diagnosis was also based on a puncture biopsy, which is necessary in destructive bone diseases suspected of spine cryptococcal infection to avoid delay in diagnosis.

Isolated cryptococcal osteomyelitis is the infection of one or more adjacent bones without extra skeletal lesions, while disseminated cryptococcal osteomyelitis is the infection of more than two non-contiguous bone sites or bone lesions with extra skeletal lesions [6, 15, 28]. Due to the difference in required treatment, differentiation between isolated and disseminated cryptococcal osteomyelitis is necessary. This can usually be achieved by fungal culture of the blood and cerebrospinal fluid, detection of cryptococcal antibodies, a brain MRI, and chest CT examination [9, 21, 24]. In our case, bacteraemia, lung and other bone infection lesions, were excluded by blood cultures, chest CT, and bone scanning. However, the patient declined lumbar puncture and brain MRI because of absence of headaches and dizziness. Furthermore, cryptococcal antibody test could not be completed due to equipment limitations. Therefore, evidence for differential diagnosis remains insufficient. Finally, considering that the patient had not experienced any central nervous system symptoms throughout the treatment, he was diagnosed with isolated cryptococcal osteomyelitis.

It is widely accepted that antifungal therapy is indispensable for the treatment of cryptococcal osteomyelitis [29, 30], but the preferred treatment approach (surgical versus nonsurgical) remains controversial. Zhou et al. [3] suggested that surgery may increase the risk of infection dissemination, and hence, did not recommend it for cryptococcal osteomyelitis, especially in immunocompromised patients. Meanwhile, nonsurgical treatment has also successfully cured some cases [13, 18, 25]. Some clinicians believe that only surgical debridement combined with antifungal treatment can achieve excellent therapeutic effect, and delayed surgery may result in a poor prognosis [6, 27]. Ruan et al. [24] and Adsul et al. [1] reported two patients with lower extremity paralysis due to cryptococcal osteomyelitis of the spine. They had a residual sensation and gait abnormality because of the delay in spinal cord decompression surgery. Nahra et al. [31] reported a 31-year-old immunocompetent woman who had necrosis of the sacral nerve root due to *Candida albicans* sacral osteomyelitis. Therefore, they advocate for early and aggressive surgery combined with long-term antifungal medication therapy for fungal osteomyelitis of the sacrum. Similar to these cases, our patient had occasional radiating pain in the legs on admission and subsequently, during the treatment, developed persistent pain and numbness. It was inferred that his sacral nerve roots were becoming affected; hence, surgical intervention was performed. Intraoperatively, we confirmed compression of the sacral nerve roots. Neither surgical nor nonsurgical treatment is universally applicable. The degree of spinal stability impairment and risk of spinal cord nerve injury is critical in deciding whether surgery is necessary for spine cryptococcal osteomyelitis. Generally, antifungal treatment alone is sufficient for cryptococcal osteomyelitis [13, 32]. However, surgical intervention may be needed in some patients with large lesions or in those at risk of vital tissue or organ damage.

In summary, we report a rare case of isolated sacrum cryptococcal osteomyelitis in an immunocompetent patient and reviewed 18 published cases of spine cryptococcal osteomyelitis. Immunocompetent individuals are also at risk for cryptococcal osteomyelitis. Due to atypical clinical symptoms and imaging findings, these alone may be insufficient for diagnosing spine cryptococcal osteomyelitis; hence, invasive examinations like puncture biopsy and fungal examinations are needed. Generally, nonsurgical therapy proves satisfactory for treatment of spine cryptococcal osteomyelitis. However, when the infective lesion is large, especially when it compresses the spinal cord and nerves, a regimen combining aggressive surgery with antifungal therapy is indispensable.

## Data Availability

The main data used or analyzed in this case report are included in this published article. More detailed data are available from the corresponding author on reasonable request.

## Abbreviations

AIDS: Acquired immune deficiency syndrome
MRI: Magnetic resonance imaging
T2WI: T2-weighted imaging
T1WI: T1-weighted imaging
CT: Computed tomography
ESR: Erythrocyte sedimentation rate
CRP: C-reactive protein
GM-test: Galactomannan detection test
